# Comparative Analysis of the IclR-Family of Bacterial Transcription Factors and Their DNA-Binding Motifs: Structure, Positioning, Co-Evolution, Regulon Content

**DOI:** 10.3389/fmicb.2021.675815

**Published:** 2021-06-10

**Authors:** Inna A. Suvorova, Mikhail S. Gelfand

**Affiliations:** ^1^Institute for Information Transmission Problems of Russian Academy of Sciences (The Kharkevich Institute), Moscow, Russia; ^2^Skolkovo Institute of Science and Technology, Moscow, Russia

**Keywords:** transcription regulation, transcription factor binding sites (TFBS), IclR-family, protein-DNA contacts, tandem binding sites, comparative genomics

## Abstract

The IclR-family is a large group of transcription factors (TFs) regulating various biological processes in diverse bacteria. Using comparative genomics techniques, we have identified binding motifs of IclR-family TFs, reconstructed regulons and analyzed their content, finding co-occurrences between the regulated COGs (clusters of orthologous genes), useful for future functional characterizations of TFs and their regulated genes. We describe two main types of IclR-family motifs, similar in sequence but different in the arrangement of the half-sites (boxes), with GKTYCRYW_3–4_RYGRAMC and TGRAACAN_1–2_TGTTYCA consensuses, and also predict that TFs in 32 orthologous groups have binding sites comprised of three boxes with alternating direction, which implies two possible alternative modes of dimerization of TFs. We identified trends in site positioning relative to the translational gene start, and show that TFs in 94 orthologous groups bind tandem sites with 18–22 nucleotides between their centers. We predict protein–DNA contacts via the correlation analysis of nucleotides in binding sites and amino acids of the DNA-binding domain of TFs, and show that the majority of interacting positions and predicted contacts are similar for both types of motifs and conform well both to available experimental data and to general protein–DNA interaction trends.

## Introduction

Interactions between DNA and proteins are crucial for many important biological processes, including replication, reparation, and the main mechanism of transcription regulation, binding of transcription factors (TFs) to specific DNA sequences (Ofran et al., 2007). Genes encoding TFs comprise a large fraction of bacterial genomes (up to 10%) (Pérez-Rueda and Collado-Vides, 2000; Rodionov, 2007), and their structure and DNA-binding specificity are often unknown (Ofran et al., 2007). Therefore, uncovering the mechanisms of protein–DNA interaction is an important problem of molecular and computational biology.

Empirical rules of the protein–DNA recognition reflect chemical and physical properties of amino acid residues and base pairs, such as their partial charge interactions, amino acid side chain flexibility, *etc.* (Lustig and Jernigan, 1995). Specific interactions with DNA are mainly formed by amino-acid side-chain atoms (Morozov and Siggia, 2007), and important and favorable contacts are usually hydrogen bonds (due to their high specificity and directional character) and acid–base interactions, since there are relatively few non-polar atoms in the DNA grooves (Seeman et al., 1976; Lustig and Jernigan, 1995; Marabotti et al., 2008), and regions of protein–DNA contacts are rich in polar residues forming electrostatic and hydrogen bonds (Gromiha and Fukui, 2011). However, other types of contacts, e.g., hydrophobic interactions, may also be important (Mirny and Gelfand, 2002), and these interaction trends are not universal, mainly since protein–DNA contacts may depend on the structural context and, in particular, on the structural family of DNA-binding proteins (Morozov et al., 2005; Rohs et al., 2010). For example, contacts between the protein and the DNA sugar-phosphate backbone presumably play a minor role in determining the specificity, but may have an influence on the positioning and orientation of TF recognition elements, thus providing a structural framework for the proper interaction (Luscombe and Thornton, 2002; Rohs et al., 2010).

Conservation of base pairs in a motif is significantly correlated with the number of contacts they make with the TF (Mirny and Gelfand, 2002; Morozov and Siggia, 2007). Base pairs forming more contacts tend to be more conserved in evolution, because these amino acid-base pair interactions may stabilize the protein–DNA complex, which makes changes in these positions detrimental (Mirny and Gelfand, 2002). Calculation of the mutual information may be used for prediction of amino acid–base contacts for particular TF families, allowing one to make structural predictions given only the sequences; such predictions can be further verified experimentally (Mirny and Gelfand, 2002; Mahony et al., 2007; Desai et al., 2009; Huang et al., 2009; Camas et al., 2010; Ravcheev et al., 2012).

### IclR-Family

The IclR-family of TFs, named after the best characterized member of the group, the glyoxylate bypass repressor in *Escherichia coli*, is a large group of proteins encountered in diverse bacteria and some archaea (Zhang et al., 2002; Yamamoto and Ishihama, 2003; Molina-Henares et al., 2006). The IclR-family includes repressors, activators, and proteins with a dual regulatory role (Molina-Henares et al., 2006; Kamimura et al., 2012; Chao and Zhou, 2013). IclR-family TFs generally regulate their own expression and expression of one or two adjacent operons (Torres et al., 2003; Yamamoto and Ishihama, 2003; Kasai et al., 2009, 2010, 2015; Schröder et al., 2012; Chao and Zhou, 2013). TFs from the IclR-family control various biological processes, such as sporulation, plant pathogenicity, quorum-sensing, biofilm formation, carbon metabolism, antibiotic production, amino acid biosynthesis and utilization, multidrug/solvent efflux (Phoenix et al., 2003; Rodionov et al., 2004; Traag et al., 2004; Molina-Henares et al., 2006; Brune et al., 2007; Yang et al., 2009; Lu et al., 2011; Schröder et al., 2012; Aguilar et al., 2014; Molina-Santiago et al., 2014; Kim et al., 2015). Most frequently they regulate the metabolism of aromatic compounds, such as catechol, homogentisate, 3-hydroxybenzoate and gentisate, 4-hydroxybenzoate and protocatechuate, 3-phenoxybenzoate, 3-(3-hydroxyphenyl)propionate, γ-resorcylate, phthalate and its isomers isophthalate and terephthalate, 2,4,6-trinitrophenol, *etc.* (Table 1). However, for many IclR-family TFs a specific function has not been determined yet (Zhang et al., 2002).

**TABLE 1:** IclR-family TFs, their functional roles and binding motifs.

**TF**	**Regulation of metabolic process**	**Binding motif**	**References**
IclR (*E. coli*)	Glyoxylate bypass	TGGAAATNATTTCCA	Pan et al., 1996; Yamamoto and Ishihama, 2003
KdgR (γ-proteobacteria)	Pectin and poly/oligogalacturonate utilization	RWWGAAACGNCGTTTCAKKA	Rodionov et al., 2004
AllR (*E. coli*)	Allantoin utilization	KTTGGAAWAWTWTTCCAAC	Rintoul et al., 2002, this study
SsgR [**Streptomyces coelicolor** A3(2)]	Sporulation	TGAAAACTCACTCCT	Traag et al., 2004, this study
HutR (*Corynebacterium resistens* DSM 45100)	Histidine utilization	GTCTGWWATWCCAGAC	Schröder et al., 2012, this study
CatR (*Rhodococcus erythropolis*)	Catechol utilization	SWWGTACGCAGAGCGTACARM	Veselý et al., 2007; Kasai et al., 2010
PcaR (*Pseudomonas putida*)	4-hydroxybenzoate, protocatechuate utilization	WWWRKTCGATWATCGSAYRRW	Romero-Steiner et al., 1994; Gerischer et al., 1998; Guo and Houghton, 1999; Kasai et al., 2010
PcaR (*Corynebacterium glutamicum*)	4-hydroxybenzoate, protocatechuate utilization	GTTCGC-N_3_-GCGAAC	Brinkrolf et al., 2006
PcaU (*Acinetobacter baylyi*)	4-hydroxybenzoate, protocatechuate utilization	TTTGTTCGATWATCGMACAMA	Gerischer et al., 1998; Popp et al., 2002; Siehler et al., 2007; Jerg and Gerischer, 2008; Kasai et al., 2010
PobR (*Acinetobacter baylyi*)	4-hydroxybenzoate, protocatechuate utilization	TTGTCCGATSATCGGACAR	Gerischer et al., 1998; Popp et al., 2002, Siehler et al., 2007; Kasai et al., 2010
HmgR (*P. putida*)	Homogentisate utilization	ATTACGTTATTCGTAAT	Arias-Barrau et al., 2004
GenR (*C. glutamicum*)	3-hydroxybenzoate, gentisate utilization	ATTCC-N_7(5)_-GGAAT	Brinkrolf et al., 2006; Chao and Zhou, 2013
MhpR (*E. coli*)	3-(3-hydroxyphenyl)propionate utilization	GGTGCACCTGGTGCACA	Torres et al., 2003
NdgR/LtbR (Actinobacteria)	Amino acid biosynthesis	KTYCRSMWYSYGRRM	Brune et al., 2007; Kim et al., 2015, this study
NpdR (*Rhodococcus opacus* HL PM-1)	2,4,6-trinitrophenol utilization	GTTCMRYATMRTGAWS	Nga et al., 2004
OphR (*Rhodococcus* sp. DK17)	Phthalate utilization	CGCGTACGCG	Choi et al., 2015
TphR (*Comamonas* sp. E6)	Terephthalate utilization	TTTTTGCGCATAGCGCAAAAA	Kasai et al., 2010
IphR (*Comamonas* sp. E6)	Isophthalate utilization	GTCTCATCAGAC and additional downstream half-site ATGGAC	Kamimura et al., 2012
PbaR (*Sphingobium wenxiniae* JZ-1T)	3-phenoxybenzoate utilization	AATAGAAAGTCTGC CGTACGGCTATTTTT	Cheng et al., 2015
TsdR (*Rhodococcus jostii* RHA1)	γ-resorcylate utilization	GTGTGRYTSSMRTCAYAC	Kasai et al., 2015

*Underlined are key positions of the group 1 consensus motif.*

Proteins from the IclR-family are ∼250 amino acid residues long and have N-terminal HTH (helix–turn–helix) DNA-binding domains and C-terminal effector-binding and multimerization domains (Zhang et al., 2002; Molina-Henares et al., 2006; Lu et al., 2010; Kamimura et al., 2012). The HTH domain is the most common and best-characterized DNA-binding motif in prokaryotes (Brennan and Matthews, 1989; Rigali et al., 2002; Ramos et al., 2005; Molina-Henares et al., 2006). It consists of an α-helix (α2), a short connecting turn, and a second α-helix (α3), often referred to as the “recognition helix,” as it directly interacts with the DNA, fitting into the major groove (Brennan and Matthews, 1989; Rigali et al., 2002; Zhang et al., 2002; Molina-Henares et al., 2006; Lu et al., 2010). Generally, HTH proteins bind as dimers to twofold symmetric DNA operator sequences, where each monomer recognizes a half-site, and IclR-family TFs are known to bind target promoters as dimers or tetramers (Rigali et al., 2002; Zhang et al., 2002; Yamamoto and Ishihama, 2003; Ramos et al., 2005; Molina-Henares et al., 2006; Lu et al., 2010).

### Structure of Binding Motifs

Different types of DNA-binding domains recognize distinct motifs, while DNA-binding proteins from the same family generally tend to recognize sites similar in length, symmetry, and specificity (Morozov and Siggia, 2007; Badis et al., 2009; Ravcheev et al., 2014; Korostelev et al., 2016; Suvorova and Gelfand, 2019). Within each family of TFs, the structure and fold of the DNA-binding domain and its mode of interaction with the binding motif are usually conserved, resulting in a certain common pattern of protein-DNA contacts (Morozov and Siggia, 2007). However, even proteins with very high (up to 60–70%) amino acid sequence identity might recognize different DNA motifs (Badis et al., 2009; Kazakov et al., 2013; Ravcheev et al., 2014).

Binding motifs have been identified for a number of IclR-family TFs, and though it is thought that there is no common consensus sequence for the entire family (Molina-Henares et al., 2006), certain types of IclR-family motifs could be distinguished. One group includes A/T-rich palindromic motifs, such as the binding motifs of IclR, KdgR, AllR, SsgR (Table 1). Another group comprises TFs with motifs with the GTNCG-N_5–6_-CGNAC consensus: HutR, CatR, PcaR, PcaU, PobR, HmgR, GenR, MhpR, NdgR/LtbR, NpdR, OphR, TphR (Table 1). Some of these motifs, namely PcaU, PobR, and HmgR, also have an additional external direct half-site repeat (Kok et al., 1998; Popp et al., 2002; Arias-Barrau et al., 2004; Molina-Henares et al., 2006; Jerg and Gerischer, 2008); and it has been shown that this direct repeat is required for the PcaU binding (Popp et al., 2002). Examples of known IclR-family TFs with motifs of the other types are IphR, PbaR, TsdR (Table 1).

### Goals

We use the comparative genomics approach to identify binding motifs and reconstruct regulons (i.e., all genes/operons regulated by a TF in a given genome) and regulogs (combined regulons of a group of orthologous TFs in different genomes) for TFs from the IclR-family. Using these data, we attempt to further characterize functional roles of IclR-family TFs, reveal tendencies in their binding site structure and localization, and predict the most favorable protein–DNA contacts.

## Materials and Methods

### Main Tools and Resources

Genomes were obtained from GenBank (Benson et al., 1999). Homologs of TFs were identified by PSI-BLAST (*E*-value cutoff, 10^–20^) (Altschul et al., 1997), and orthologs were identified by construction of phylogenetic trees for identified homologs and analysis of their genomic context (e.g., co-localization with genes of a certain metabolic pathway in most genomes). The genomic context was analyzed using MicrobesOnline (Dehal et al., 2010).

Amino acid and nucleotide sequence alignments were performed using the MAFFT service (default parameters) (Katoh et al., 2019). Phylogenetic trees were built using PhyML (default parameters) (Dereeper et al., 2008) and visualized with Dendroscope (Huson et al., 2007).

Motif logos were constructed using WebLogo (Crooks et al., 2004).

Molmil was used for the visualization of PDB data (Bekker et al., 2016).

### Phylogenetic Footprinting

Candidate binding sites were identified (or confirmed if they have been previously predicted) by phylogenetic footprinting (Rodionov, 2007). We manually analyzed alignments of upstream regions of orthologous genes presumably belonging to the respective regulon (genes encoding TFs, as they are often auto-regulated, and genes co-localized with them) (Gelfand et al., 2000; Tan et al., 2005; Martínez-Antonio et al., 2008) and identified consecutive conserved nucleotides, relying on the assumption that binding sites are more conserved than surrounding intergenic regions. These conserved regions, i.e., predicted binding sites, were then used as training sets for construction of nucleotide position weight matrices (PWMs) for each TF by the SignalX program as previously described (Gelfand et al., 2000). PWMs were then used for exhaustive scan of genomes possessing the corresponding TFs in search for additional candidate binding sites (and regulon members) as described further.

All identified binding sites are given in Supplementary Table 1, spreadsheets “group 1” and “group 2.”

### Reconstruction and Analysis of Regulons and Regulogs

Computational search for candidate binding sites in upstream gene regions [400 nucleotides (nt) upstream and 50 nt downstream relative to the annotated translational gene start] was performed using the built PWMs and the GenomeExplorer program package (Mironov et al., 2000). Score thresholds for the identification of sites were selected so that candidate sites upstream of functionally relevant genes were accepted, while the fraction of genes preceded by candidate sites did not exceed 5% in studied genomes. Weaker sites (with scores 10% less than the threshold) were also accepted if their positions were similar to positions of stronger sites upstream of orthologous genes and there were no stronger competing sites in the intergenic region. New candidate members were assigned to a regulon if they were preceded by candidate binding sites in several genomes, the exact number of genomes depending on the number of sequenced genomes in a taxonomy unit. The reconstructed regulons were extended to include all genes in putative operons, the latter defined as the strings of genes transcribed in the same direction, with intergenic distances not exceeding 200 nt, when such organization persisted in several genomes. All genes comprising putative regulated operons were included into functional analysis of regulons.

To analyze positioning of sites relative to gene start, coordinates of site centers were calculated to account for differences in the motif lengths. In case of even-length sites, coordinates of site centers were rounded to the smaller whole number. The relevant data are given in Supplementary Table 2.

Content of the studied regulogs (combined regulons for each orthologous group of TFs) was analyzed using the BiBit algorithm for biclustering^1^ of data reflecting regulatory interactions, in order to reveal frequently co-regulated genes and to identify orthologous groups of TFs most similar in the regulog composition. Only genes with unambiguously assigned COG (clusters of orthologous genes, Galperin et al., 2019) or PFAM IDs were considered in this analysis. The data are given in Supplementary Table 3.

### Correlation Analysis

We restricted our study to IclR-family TFs (COG1414) from completely sequenced genomes present in the MicrobesOnline database (Supplementary Table 1, spreadsheet “list of genomes”). Only TFs predicted to have palindromic binding motifs satisfying either of two identified IclR-family consensuses were selected for the correlation analysis. Correlations were calculated between amino acid residues of DNA-binding HTH domains and nucleotides in binding sites, regions with gaps were cut out of the amino acid alignments (Supplementary Table 1, spreadsheets “HTH alignment group 1,” “HTH alignment group 2”), positions of amino acids were subsequently re-numbered starting from the beginning of the HTH domain, counting from zero.

Structural data of TtgV from *P. putida* in complex with DNA was taken as a reference model (Lu et al., 2010), supported by data on DNA-binding of wild-type and mutant TtgV and PobR (Kok et al., 1998; Fillet et al., 2009; Molina-Santiago et al., 2014), as well as structural data and DNA-binding modeling of TM0065 from *T. maritima* (Zhang et al., 2002).

Correlations were calculated using the Prot-DNA-Korr program package (Korostelev et al., 2016). The program calculates the correlation between each pair of columns, one from the amino acid alignment of the HTH domains, the other from the nucleotide alignment of the sites. Even-length and odd-length sites were aligned using central gap insertions, differences in sites length were compensated by introducing gaps on flanks. Datasets used in this work are given in Supplementary Table 1, spreadsheets “group 1” and “group 2.” The mutual information was used as a measure of correlation. The statistical significance value of the mutual information was calculated as the *Z*-score. Correlated pairs of positions were displayed as a heatmap, with the color denoting statistical significance (significant correlations colored in the red-yellow palette), and as contingency tables (Supplementary Table 4) containing expected and observed counts of amino acid-nucleotide pairs, as well as χ^2^ scores (scores higher than 30 were considered significant, scores higher than 50 were considered as strong preferences or avoidances, depending on the corresponding expected and observed values). For additional details about Prot-DNA-Korr see http://bioinf.fbb.msu.ru/Prot-DNA-Korr/main.html.

## Results

### Binding Sites, Structure and General Statistics

Four thousand eight hundred and nine candidate binding sites have been predicted for 1340 IclR-family TFs constituting 181 orthologous groups in 320 bacterial genomes (Supplementary Table 1). Binding sites were identified via phylogenetic footprinting and further scanning of genomes with PWMs built based on footprinting results, as described in Materials and Methods. For verification of our results we used previously published experimental and comparative data (summarized in Table 1), as well as independently obtained data on candidate binding sites of many IclR-family TFs, available in the RegPrecise database^2^, and observe a complete agreement with it. Still, many TFs studied in this work are novel.

We have observed that identified IclR-family palindromic binding sites fall into either of two main types, with GKTYCRYW_3–4_RYGRAMC (group 1) or TGRAACAN_1–2_TGTTYCA (group 2) consensuses.

**FIGURE 1:**
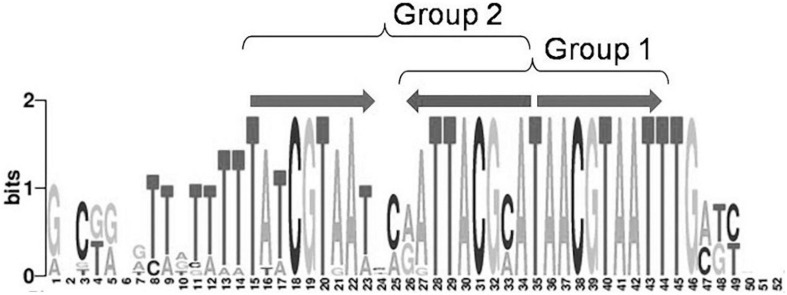
Example of a IclR-family three-box binding motif matching both group 1 and group 2 consensuses.

Moreover, 32 orthologous groups comprising 199 TFs have been predicted to bind three-box binding sites, with one pair of boxes corresponding to the first variant of the IclR-family motif consensus, and the other pair, to the second consensus variant (Figure 1). This feature may indicate a possibility of alternative dimerization modes of some IclR-family TFs, and agrees well with previous data on PcaU from the IclR-family, which has a three-box binding motif where the additional third box is also required for the binding of TF (Popp et al., 2002). As sites matching either the first or the second type of the consensus (group 1 and group 2) were analyzed separately, TFs of these 32 orthologous groups (cells marked orange in Supplementary Tables 1,3) were considered in both groups 1 and 2, excluding either the first or the third box.

Taking that into account, we have analyzed 3932 predicted sites for 1257 IclR-family TFs that match consensus GKTYCRYW_3–4_RYGRAMC (group 1), and 877 sites for 282 IclR-family TFs with consensus TGRAACAN_1–2_TGTTYCA (group 2).

**FIGURE 2:**
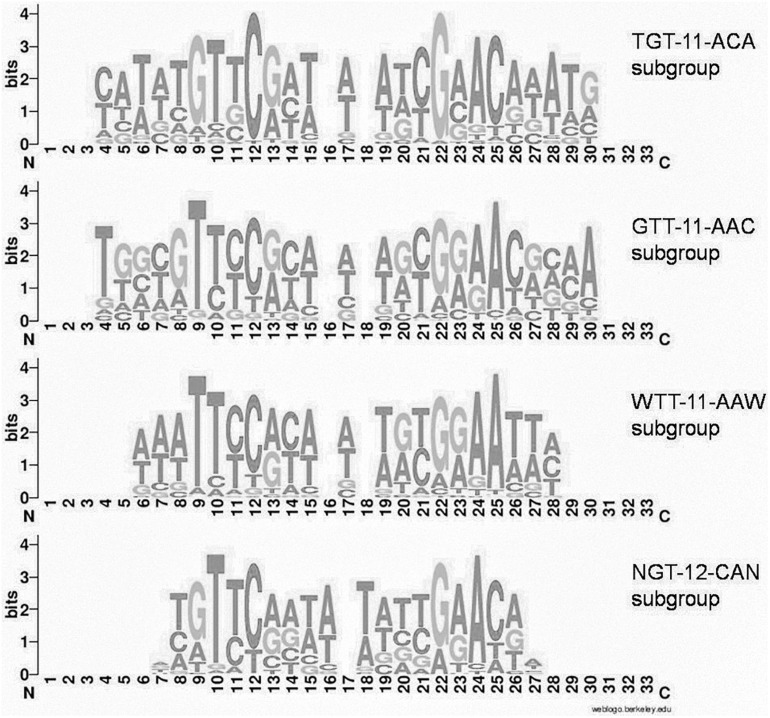
Joint LOGO diagrams of aligned binding sites of group 1 subgroups. Gaps are inserted to align even and odd binding sites.

Group 1 of motifs is more prominent and comprises four main variants (subgroups) with differences in some peripheral positions (Figure 2):

(i) TGTYCRYW_3_RYGRACA (41 orthologous groups, 283 TFs, 981 sites, further denoted TGT*–*11*–*ACA)

(ii) GTTYCRYW_3_RYGRAAC (54 orthologous groups, 427 TFs, 1364 sites, further denoted GTT*–*11*–*AAC)

(iii) WTTYCRYW_3_RYGRAAW (43 orthologous groups, 303 TFs, 935 sites, further denoted WTT*–*11*–*AAW)

(iv) NGTYCRAW_4_TYGRACN (24 orthologous groups, 199 TFs, 517 sites, further denoted NGT*–*12*–*ACN).

There is no apparent correlation between the motif structure and phylogeny, all types of motifs (groups and subgroups) are scattered along the phylogenetic tree (data not shown).

IclR-family TFs are present predominantly in Proteobacteria and Actinobacteria, and we have observed some differences in the taxonomic distribution among the motif groups and subgroups (Table 2). For example, in Firmicutes GTT*–*11*–*AAC type motifs are overrepresented and the TGT*–*11*–*ACA type absent, Proteobacteria have weaker representation of GTT*–*11*–*AAC motifs, compared to other subgroups, and only group 2 type motifs are identified in Thermotogales.

**TABLE 2:** Taxonomic distribution of motif groups and subgroups.

**Taxonomic group\Motif type**	**TGT-11-ACA (group 1)**	**GTT-11-AAC (group 1)**	**WTT-11-AAW (group 1)**	**NGT-12-CAN (group 1)**	**Group 1, total**	**Group 2, total**
Alphaproteobacteria	27.69%	15.00%	19.35%	19.53%	17.48%	20.81%
Betaproteobacteria	29.23%	17.73%	21.94%	21.09%	15.86%	20.81%
Gammaproteobacteria	21.54%	11.82%	29.68%	19.53%	21.04%	30.06%
Delta-Epsilonproteobacteria	0.00%	1.36%	0.00%	2.34%	2.27%	1.16%
Acidithiobacillia	0.00%	0.00%	0.65%	0.00%	0.32%	0.00%
Actinobacteria	20.00%	30.45%	20.00%	28.13%	22.98%	10.40%
Firmicutes	0.00%	18.18%	4.52%	6.25%	13.92%	10.98%
Deinococcus-Thermus	1.54%	1.82%	3.23%	0.00%	2.27%	0.00%
Chloroflexi	0.00%	2.73%	0.00%	0.00%	1.94%	0.00%
Fusobacteria	0.00%	0.91%	0.00%	1.56%	0.97%	0.58%
Bacteroidetes/Chlorobi	0.00%	0.00%	0.65%	1.56%	0.97%	1.16%
Thermotoga	0.00%	0.00%	0.00%	0.00%	0.00%	3.47%
Dictyoglomi	0.00%	0.00%	0.00%	0.00%	0.00%	0.58%

### Binding Sites Positioning

We have analyzed not only structure, but also localization of identified sites relative to translational gene start in order to see whether there are any apparent tendencies in site positioning.

**FIGURE 3:**
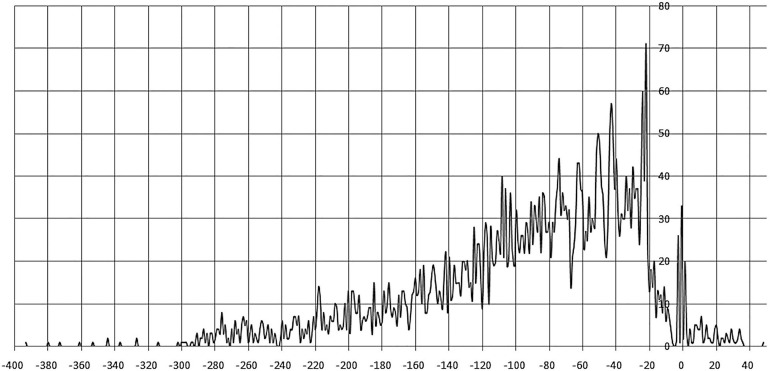
Positioning of IclR-family binding sites. Horizontal axis – the distance between the site center and the gene start; vertical axis – the number of binding sites.

Sites centered at –400 to –300 nt and +40 to +50 regions are very rare. The most frequent position of site centers is –22 nt, a prominent peak is also observed at –3 to +1 nt. The majority of site centers are localized from –20 to –80 nt upstream of the gene start, gradually decreasing up to –300 nt. Similar trends in site localization were previously observed for other TF families, e.g., LacI (Ravcheev et al., 2014). Moreover, in this 60–nt zone we observe prominent oscillations in positioning of sites, with the distance between both pronounced peaks and minima approximately equal to one DNA turn (Figure 3 and Supplementary Table 2).

**FIGURE 4:**
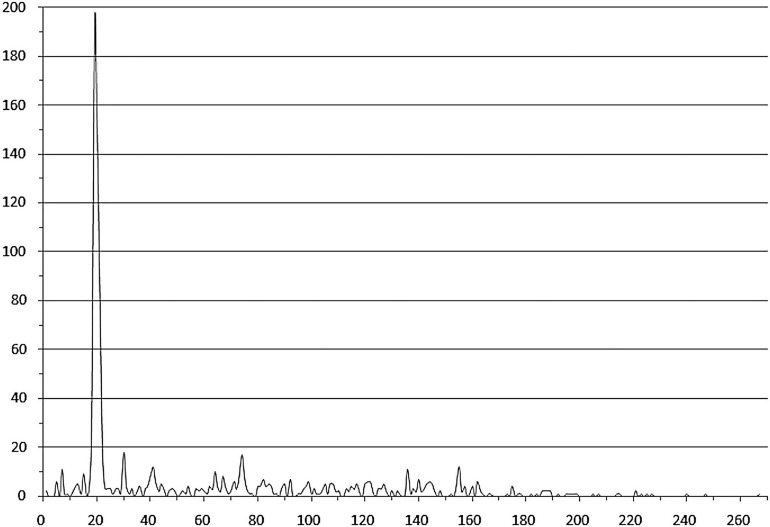
Distances between tandem binding sites. Horizontal axis – the distance between the adjacent site centers; vertical axis – the number of site pairs. Duplicates corresponding to divergently transcribed regulated genes are removed.

Notably, many studied TFs (from 94 orthologous groups, marked with italics in Supplementary Table 3) in both group 1 and group 2 are predicted to bind tandem palindromic sites with inter-site distance (between the site centers of symmetry) of 18–22 nt (mainly 19–21 nt, that is approximately two DNA turns). This observation agrees with the known fact that IclR-family TFs can bind DNA as tetramers (Molina-Henares et al., 2006; Lu et al., 2010), with dimers facing the same side of DNA. The inter-site distances of approximately three and four DNA turns (possibly also allowing for the cooperative binding) are also overrepresented, but this trend is less pronounced (Figure 4 and Supplementary Table 2).

### Regulog Content

Identification of candidate binding sites allows for reconstructing regulons and regulogs of corresponding TFs. We analyze content of IclR-family regulogs, speculating about functional characterizations of TFs and their regulated genes.

Regulogs of the studied IclR-family TFs vary in size, from 1 to 55 regulated COGs in a regulog. Most regulogs are rather small, more than half of them (98 out of 181) comprise ten or less regulated COGs.

The regulog content also differs widely between orthologous groups: out of 631 identified COGs, just 300 were present in two or more regulogs, and only 48 COGs, in ten or more regulogs (Supplementary Table 3, spreadsheet “table COGs summary”). Out of these 48 “top” COGs, many are potentially involved in the metabolism and transport of aromatic compounds and sugars or sugar acids (Table 3). We observe difference in the distribution of these COGs in motif groups and subgroups. COGs involved in metabolism and transport of sugars and sugar acids are underrepresented in regulons of TGT-11-ACA and WTT-11-AAW motif types compared to other subgroups, while COGs involved in the metabolism and transport of aromatic compounds are overrepresented in regulons of TGT-11-ACA type. On the contrary, COGs involved in the metabolism and transport of sugars and sugar acids are frequently present in regulons of GTT-11-AAC and NGT-12-ACN motif types, while many COGs involved in aromatic metabolism are underrepresented in these subgroups. Moreover, COG1028 and COG1012 encoding dehydrogenases are especially overrepresented in NGT-12-ACN motif subgroup (Table 3).

**TABLE 3:** Distribution of main COGs in regulogs for motif groups and subgroups.

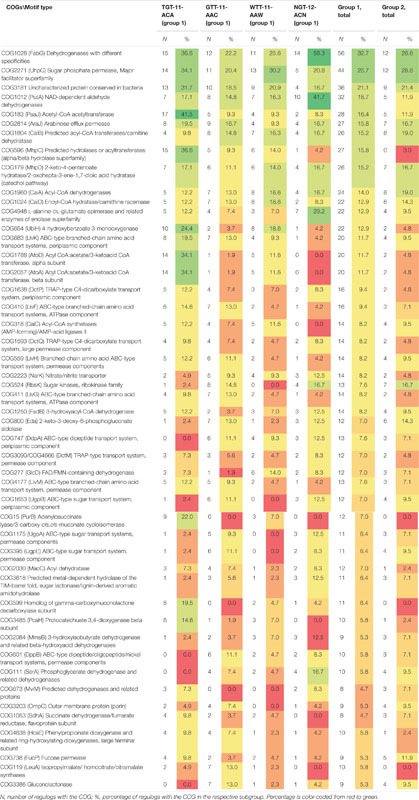

*N, number of regulogs with the COG; %, percentage of regulogs with the COG in the respective subgroup. Percentage is color-coded from red to green.*

We also attempted to study the COGs co-occurrence patterns in IclR-family regulogs to reveal possible functional connections between them, especially important for poorly characterized COGs, and it might help in understanding metabolic functions of IclR-family TFs.

As the composition of IclR-regulogs widely varies, we do not see co-occurences of COGs throughout the majority of regulogs, but observe a number of cases, where COGs are present in a small fraction of regulogs, but if present, are always or almost always found together. The most frequently associated pair is COG1788 (AtoD) and COG2057 (AtoA), each one of them is present only in 20 regulogs out of 181, but they co-occur in all of these twenty (for other examples, see Supplementary Table 3, spreadsheet “most frequent association”).

In addition to obvious co-occurrence of transporter subunits (COGs LivKHMGH, DctPQM, DdpA-DppBCD, UgpABE, HisJM-GlnQ, TauABC, AraH-MglA-RbsB *etc.*) and enzyme subunits (e.g., AtoDA), we identified other frequently co-regulated COGs in IclR-family regulogs (Supplementary Table 3). A large group of COGs, known or presumed to be involved in the metabolism of aromatic compounds, co-occurs in many regulogs in various combinations, with three subsets of the most frequently associated COGs (Supplementary Table 3, spreadsheet “sets of COGs”) being:

(i) COGs involved in the degradation of benzoate, catechol, muconate and their derivatives via the ortho-cleavage pathway, channeling them into the TCA cycle through β-ketoadipate, and COGs that likely play a role in transport of aromatic compounds (Li et al., 2010; Suvorova and Gelfand, 2019);

(ii) COGs forming the meta-cleavage degradation pathway of benzoate, catechol and their derivatives, COGs that may be involved in the degradation of aromatic compounds through CoA thioesters and forming the downstream part of the β-ketoadipate pathway, and COGs that may be involved in transport of aromatic compounds (Arai et al., 2000; Zaar et al., 2001; Gescher et al., 2002; Suvorova and Gelfand, 2019);

(iii) COGs likely involved in the quinate/shikimate and 4-hydroxyphenylpyruvate metabolism, and transport of aromatic compounds.

One more set is comprised of COGs involved in the metabolism and transport of sugars and sugar acids and/or aromatic compounds (Hobbs et al., 2012, 2013; Maruyama et al., 2015; Suvorova and Gelfand, 2019; Watanabe et al., 2020), and includes two most frequently associated subsets (Supplementary Table 3, spreadsheet “sets of COGs”).

Thus, IclR-family TFs indeed regulate mainly metabolism and transport of various aromatic compounds. Analysis of frequently associated COGs may be useful not only for functional characterization of unknown TFs, but also COGs with unknown or insufficiently studied functions; examples of such frequently associated COGs identified in this study are COG3254 and COG3618, as well as functional association of COG1545, COG2030, COG3181, and COG3333 with genes of metabolism and transport of aromatic compounds (Supplementary Table 3, spreadsheet “sets of COGs”).

### Protein–DNA Correlations

One of the goals of this work was to find correlations between nucleotides of identified binding sites and amino acid residues of DNA-binding HTH domains of the IclR-family TFs to predict potential protein–DNA contacts. Correlations, calculated based on mutual information, were found using the Prot-DNA-Korr program package as described in section “Materials and Methods.”

#### Group 1

**FIGURE 5:**
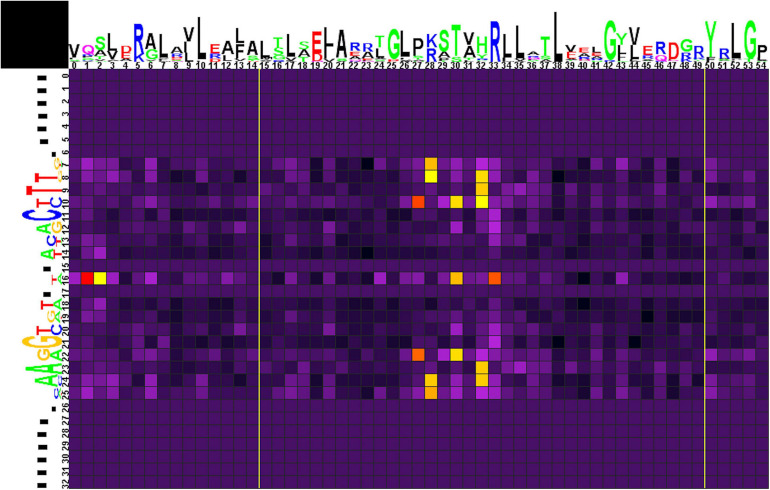
Heat map of correlations between amino acids and nucleotides for group 1 TFs and their binding sites. Sequence logos of HTH DNA-binding domains and their binding sites are shown on the top and to the left of the heat map, respectively. The total height of symbols at each position reflects the positional information content, whereas the height of individual symbols is proportional to the positional amino acid or nucleotide frequencies. The correlation scores are color-coded from yellow to red for amino acid-nucleotide pairs with statistical significance of correlation exceeding an automatically defined threshold (with red assigned for the most correlated pair). The violet-black palette is used for other pairs. Yellow lines denote positions where gaps have been removed from the amino acid alignment.

The correlation analysis for group 1 (Figure 5 and Supplementary Table 4, spreadsheet “group 1”) shows that amino acids at positions 1, 2, 30, and 33 of the HTH domain correlate with nucleotides in the central position 16 of the binding motif. The central A/T pair of odd-length motifs is weakly avoided by Pro at position 1 of the HTH domain. Even-length motifs (with a central gap in the alignment) show a strong preference for Pro, weakly prefer Lys, and strongly avoid Gln at position 1; weakly prefer Ala and Pro at position 2; strongly prefer Ser and, more weakly, Glu at position 30; and strongly prefer Ala, Asn, and weakly prefer Asp, Glu, Gly, His, Leu, and strongly avoid Arg at position 33 of the HTH domain.

Amino acid residues at positions 27 and 30 of the HTH domain are correlated with nucleotides at positions 10/22 of the binding motif. Strong preference here is seen for His27 with the G/C pair and for Lys30 with A/T. Amino acid residues at position 28 correlate with nucleotides at positions 7/25 and 8/24. Lys28 strongly prefers G/C and avoids A/T at positions 7/25, and Leu28 is weakly correlated with C(7). Arg28 strongly prefers G/C and avoids A/T at positions 8/24. Amino acids at position 32 correlate with nucleotides at positions 8/24, 9/23, and 10. Similar to Arg28, Arg32 strongly prefers G/C and avoids A/T at positions 8/24 and, weaker, prefers G(10). Gln32 shows strong preference toward A/T (9/23), while His32 weakly prefers C(10).

Correlations of Asp28, Glu28, Gln32 with gaps at positions 8/24 are caused by flanking gaps inserted due to differences in sites lengths and are not considered further.

We also performed correlation analysis separately for each of four subgroups of group 1, to identify their contribution to the results of the whole group, and also for variants (i)–(iii) combined, to assess the differences between the odd-length and even-length motifs (results given in Supplementary Table 4 and Supplementary Figures 1–5).

The data on all groups and subgroups (see below) are summarized in Supplementary Table 4, spreadsheet “table of correlations, summary.”

#### Group 2

**FIGURE 6:**
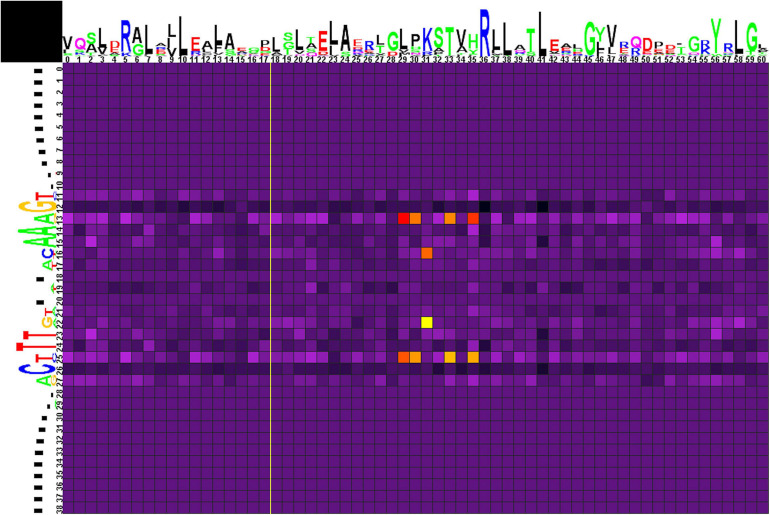
Heat map of correlations between amino acids and nucleotides for group 2 TFs and their binding sites. Notation as in Figure 5.

Correlation analysis for group 2 of motifs (Figure 6 and Supplementary Table 4, spreadsheet “group 2”) reveals multiple correlations of nucleotides at positions 13/25 with amino acids in positions 29, 30, 33, and 35. Here, Glu29 and Lys33 strongly prefer A/T, Leu35 and, more weakly, Val29 and His30 prefer G/C. Amino acid residues at position 31 are correlated with nucleotides at positions 16/22, although without significant preference for specific protein–DNA contacts.

### Main Predicted Protein–DNA Contacts

The analyzed binding motifs have a symmetrical palindromic structure, hence, the obtained heat maps are also mainly symmetrical. If not, in most cases the correlation with the symmetrical base pair is only slightly lower than the significance threshold, although the same trend is still observed; however, in other cases there might indeed be asymmetry in the dimer/tetramer structure of a TF, as shown, e.g., for TtgV (Lu et al., 2010). Due to the symmetry, the observed correlations are by default shown for either a G/C or an A/T pair. Further differentiation between the contribution of G and C, or A and T, is not always possible, and may require additional information, e.g., donor–acceptor properties of the interacting amino acids *etc*. Generally, hydrogen-bond donor residues (Arg, His, Lys, Ser, Thr) are known to bind G; hydrogen-bond acceptor residues (Asp, Glu) prefer C; while Asn and Gln, that can act both as donors and acceptors, prefer A (Seeman et al., 1976; Marabotti et al., 2008).

Taking into account only strong significant correlations, i.e., excluding weak ones, as well as amino acid positions associated with gaps (Supplementary Table 4, spreadsheet “table of correlations, summary”), chemical properties of amino acids residues and nucleotide bases suggest the following main predicted protein–DNA contacts for group1 and its subgroups:

• Thr5 and Glu26 with A/T (10/22) for the WTT–11–AAW subgroup;
• His27 with G/C (10/22) for group 1 with the contribution of all odd-length motif subgroups, mainly the GTT–11–AAC subgroup, the likely contact is His–G;
• Lys28 with G/C (7/25) for group 1 with the contribution of all odd-length motif subgroups, mainly the TGT–11–ACA subgroup, the likely contact is Lys–G;
• Arg28 and Arg32 with G/C (8/24) for group 1, the latter with the contribution of all odd-length motif subgroups, the likely contacts are Arg–G;
• Arg28 with G/C (9/23) and Arg32 with G9 for the GTT–11–AAC subgroup, the likely contacts are Arg–G;
• Ile28 with G9 for the GTT–11–AAC subgroup;
• Lys30 with A/T (10/22) for group 1 with the main contribution of the WTT–11–AAW subgroup;
• Gln32 with A/T (9/23) for group 1 with the contribution of all odd-length motif subgroups, mainly GTT–11–AAC and WTT–11–AAW, the likely contact is Gln–A;
• Gly32 with G(10) for the NGT–12–ACN subgroup;
• Gly33 and Glu33 with G/C (11/21), and Ala33 with T21 for all odd-length motif subgroups, with the main contribution of GTT–11–AAC subgroup, the likely contact is Glu–C;
• Pro33 with G/C (11/21) for all odd-length motif subgroups, with the main contribution of WTT–11–AAW subgroup.

The main predicted protein–DNA contacts identified for group 2 are (Supplementary Table 4):

• Glu29 and Lys33 with A/T (13/25)
• Leu35 with G/C (13/25).

## Discussion

### Comparison of the Group 1 and Group 2 Motifs

**FIGURE 7:**
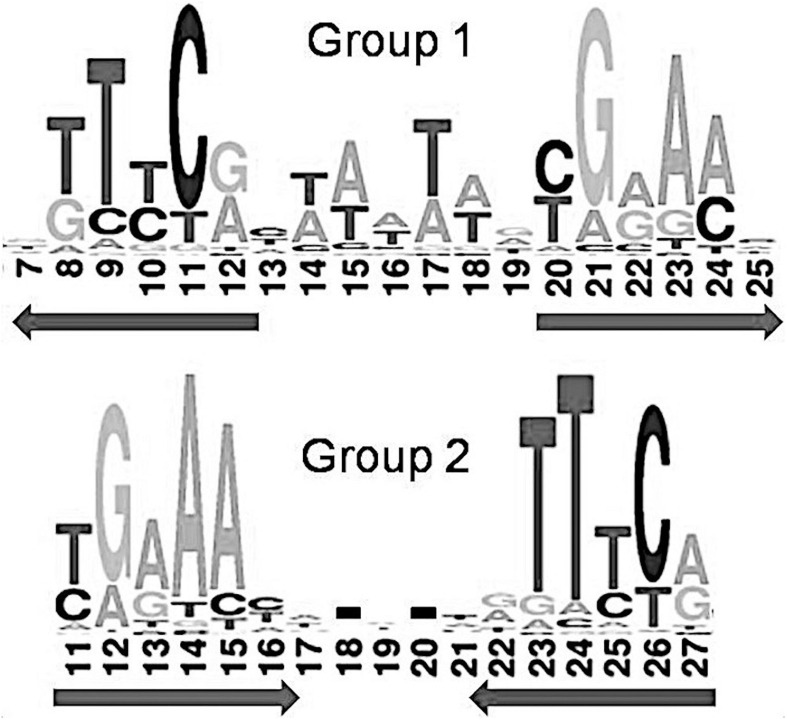
Joint LOGO diagrams of all group 1 and group 2 aligned binding sites. Arrows denote similar palindromic parts.

Previous comparison of binding motifs for some individual TFs revealed no common consensus for the IclR-family and no distinct similarity (Molina-Henares et al., 2006), especially between motifs that fall in group 1 and group 2 by our classification. Here, using a large collection of identified binding sites, we not only specify two main types of IclR-family motifs, but also find that they are similar in sequence but differ in the arrangement of half-sites of the palindrome. Joint LOGO diagrams of aligned binding sites from each group clearly show that the left half of the group 1 binding motifs corresponds to the right half of the group 2 binding motifs, and vice versa (Figure 7). It implies that there could be two different modes of dimerization of the IclR-family TFs. Moreover, since we have identified IclR-family binding motifs comprised of three boxes with alternating direction, where one pair of boxes corresponds to the group 1 consensus, and the other pair of boxes, to the group 2 consensus, we may assume that some IclR-family TFs are capable of alternative dimerization, possibly providing for more variable and precise regulation of transcription. It can be experimentally validated, for example, via DNA footprinting to precisely identify protected and sensitive regions upstream of the regulated genes and SPR to examine protein–DNA interaction.

This observation is supported by previous studies of IclR-family TFs PcaU, PobR, and HmgR, for which three-box binding motifs have been identified (Kok et al., 1998; Popp et al., 2002; Arias-Barrau et al., 2004; Molina-Henares et al., 2006; Jerg and Gerischer, 2008).

Despite differing organization of the boxes, their sequence similarity allows us to indirectly compare the predicted protein–DNA contacts for group 1 and group 2. Nucleotides 13/25 and 16/22 of the group 2 binding motifs, involved in the protein–DNA interaction according to the correlation analysis, correspond to nucleotides 10/22 and 7/25 of the group 1 motifs, respectively (Figure 7). Due to the differently removed gaps in the alignment, amino acid positions 29, 30, 31, 33, 35 of the aligned HTH domains of the group 2, which interact with DNA according to the correlation data, correspond to amino acid positions 26, 27, 28, 30, 32 of the group 1 HTH domains, respectively. Taking all this into account, we see quite congruent predictions for protein–DNA contacts in both groups (Table 4). Thus, despite the different structure of binding motifs, the interaction of TFs with DNA likely has similar features throughout the whole IclR-family.

**TABLE 4:** Comparison of predicted group 1 and group 2 protein–DNA contacts.

**Group 1**			**Group 2**		
**Amino acid**	**Nucleotides**	**Predicted contacts**	**Amino acid**	**Nucleotides**	**Predicted contacts**
26	10/22	**Glu-A/T**^a^, Trp-G/C^a^	29	13/25	**Glu-A/T**, Val-G/C
27	10/22	**His-G/C**	30	13/25	His-G/C
28	7/25	**Lys-G/C**, Leu-C	31	16/22	Not significant correlation
30	10/22	**Lys-A/T**	33	13/25	**Lys-A/T**
32	10/22	Arg-G, His-C, **Gly-G**/C^b^, Tyr-G/C^b^	35	13/25	**Leu-G/C**

*^*a*^Only in subgroup (iii). ^*b*^Only in subgroup (iv). Bold font denotes strong preferences. Differences in numbering between group 1 and group 2 are due to removal of gaps from the alignment (see Supplementary Table 1, spreadsheets “HTH alignment group 1,” “HTH alignment group 2”).*

### Protein–DNA Contacts

The main goal of this study was to predict protein–DNA contacts for IclR-family TFs via correlation analysis. In order to validate these predictions, we compared the observed correlations with known data on protein–DNA interactions of TFs from the IclR-family (summarized in Table 5).

**TABLE 5:** Congruence of the correlation analysis results and data on experimentally identified and predicted protein–DNA interactions of TtgV, PobR, and TM0065.

**Equivalent amino acid positions**				
**Group 1 and/or subgroups**	**Group 2**	**TtgV**	**PobR**	**TM0065**

1^a^	1	Gln15	Ala24	Asn2^b^
2^a^	2	Val16	Gly25	Thr3^b^
5^a^	5	Arg19^b^	Lys28	Lys6^b^
27^a^	30^a^	Pro46^b^	Ser55	Ser33
28^a^	31^a^	Arg47^b^	Arg56^b^	Val34
29^a^	32	Ser48^c^	Thr57^b^	Ser35^b^
30^a^	33^a^	Thr49^c^	Ala58	Asn36
32^a^	35^a^	Gln51^c^	Arg60^b^	Tyr38
33^a^	36	Arg52^c^	Arg61^b^	Lys39

*^*a*^Positions with identified correlations. ^*b*^Positions experimentally found to be critical for DNA binding/TF functioning (for TtgV and PobR) or predicted to be critical (for TM0065). ^*c*^Positions forming direct specific protein–DNA contacts. Differences in numbering between group 1 and group 2 are due to removal of gaps from the alignment (see Supplementary Table 1, spreadsheets “HTH alignment group 1,” “HTH alignment group 2”).*

One 3D structure of the IclR-family TF in complex with DNA is currently available, namely, TtgV from *P. putida* (PDB:2XRO), a regulator of the RND-family efflux transporters (Lu et al., 2010). According to the crystal protein–DNA structure, residues Ser48, Thr49, Gln51, and Arg52 of the recognition helix of the HTH domain directly and specifically interact with the DNA major groove (Lu et al., 2010). These observations are supported by results of mutational analysis and data on other IclR-family TFs.

For example, experiments on wild-type and mutant TtgV show that residues from Arg47 to Ile54, Leu57, Glu60, and Phe61 are involved in binding DNA (Fillet et al., 2009; Molina-Santiago et al., 2014). Similarly, in mutant PobR from *Acinetobacter baylyi* ADP1, Arg56, Thr57, Lys64, Lys67 (corresponding to Arg47, Ser48, Asn55, Glu58 of TtgV, respectively) are important for DNA binding; mutants in Arg60 and Arg61 (Gln51 and Arg52 of TtgV) fail to grow on the PobR inducer, 4-hydroxybenzoate (Kok et al., 1998). It also agrees with the prediction that Glu25, Ser35, Met41, and Leu44 in TF TM0065 from *Thermotoga maritima* (respectively, Ala38, Ser48, Ile54, and Leu57 of TtgV) mediate binding specificity due to their high conservation in the α2 and α3-helices (Zhang et al., 2002).

The N-terminal end of the α1-helix likely can contact the minor groove, and thus Asn2, Thr3, Lys5, Lys6 in TM0065 (Gln15, Val16, Ala18, Arg19 in TtgV) have been predicted to form contacts with DNA and affect specificity (Zhang et al., 2002).

TtgV mutants at Arg19, Ser35, and Gly44 fail to bind DNA; moreover, residues equivalent to Pro46 are highly conserved on the multiple alignment of IclR-family TFs, and thus also are likely important for DNA binding (Lu et al., 2010; Molina-Santiago et al., 2014). Residues Arg19 and Ser35 lie across the minor grooves and interact with the DNA phosphate backbone, which is possible due to the strong bending of the operator sequence bound to TtgV, and residues Gly44 and Pro46 within the turn of the HTH domain are involved in this distortion, hence playing a role in the DNA binding (Lu et al., 2010; Molina-Santiago et al., 2014). Glycine residues may not interact directly with DNA, but provide flexibility to bind DNA targets with different half-site spacing (Zhang et al., 2002; Molina-Henares et al., 2006).

**FIGURE 8:**
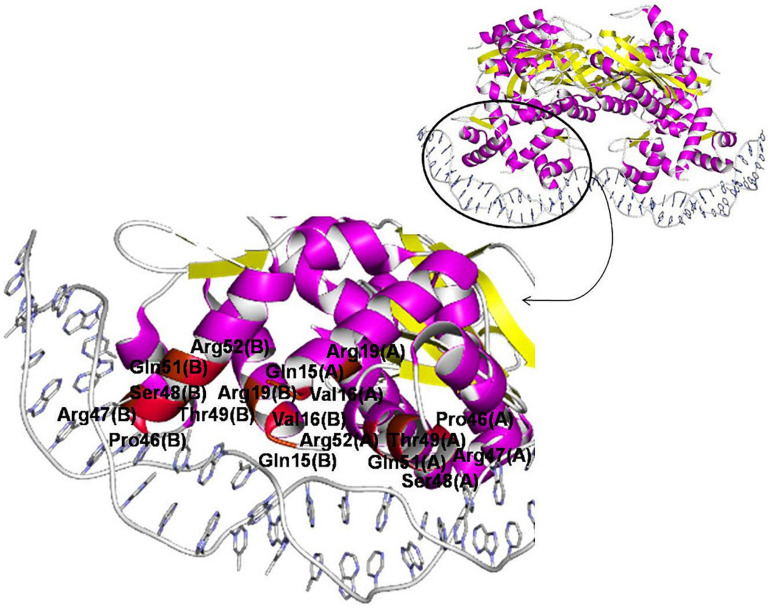
Amino acid positions involved in protein–DNA interaction of IclR-family TFs. TtgV in complex with DNA (PDB:2XRO) is chosen for visualization. Positions, for which protein–DNA correlations congruent with previous experimental and comparatively predicted data (see Table 5) are found, colored with red (even numbers) and brown (odd numbers).

The correlation analysis shows that most of the predicted contacts with DNA in all studied groups and subgroups are formed by the α3-helix of the HTH domain and, less frequently, the N-terminal part of the α1-helix, and the majority of amino acid positions significantly correlated with site positions and likely responsible for the binding specificity (nine out of 12 positions for group 1 and/or its subgroups, and four out of five positions, for group 2) correspond well to those previously identified for TtgV and PobR, and predicted for TM0065 (Figure 8, Table 5, and Supplementary Table 4, spreadsheet “table of correlations, summary”) (Kok et al., 1998; Zhang et al., 2002; Fillet et al., 2009; Lu et al., 2010; Molina-Santiago et al., 2014). Our results also agree with previous studies, where it has been demonstrated that residues of the recognition helix of the HTH domain form most of the protein–DNA contacts predicted via the correlation analysis (Korostelev et al., 2016; Suvorova and Gelfand, 2019).

It has been previously claimed that Arg, Asn, Lys, Gln, Thr, Ser, Asp, and Gly account for more than 70% of protein–DNA contacts, with Lys and Arg frequently dominating in the interactions (Molina-Henares et al., 2006), and Arg alone accounts for 23% of contacts (Marabotti et al., 2008). This trend has been observed in this study as well. The majority of predicted interactions involved these amino acids: one out of one of strong correlations in subgroups (i) and (iv), four out of eight in subgroup (ii), three out of five in subgroup (iii), five out of six if the entire group 1 is considered, and one out of three in group 2. Arg and Lys are among the most frequent ones: combined, they account for four out of six strong correlations in group 1, and one out of three in group 2 (Supplementary Table 4, spreadsheet “table of correlations, summary”).

Arg–G, Asn–A, Asp–C, Gln–A, Glu–C, Lys–G, and, to a lesser extent, His–G and Ser–G, appear to be the most relevant, strongest and highly specific contacts (Lustig and Jernigan, 1995; Marabotti et al., 2008). Preferences are also known for Ala–C, Cys–G, Gly–G, Leu–A, Thr–G, and Trp–C (Marabotti et al., 2008). Many of protein–DNA contacts predicted for the IclR-family TFs conform well to these preferences, e.g., five out of six strong correlations in group 1 (Supplementary Table 4).

## Conclusion

We have identified regulated genes and binding sites for 1340 IclR-family TFs from 181 orthologous groups in 320 bacterial genomes. Despite the prevalent opinion that IclR-family motifs have no common consensus, here we describe two main types of IclR-family motifs, similar in sequence but different in the arrangement of the boxes. This, together with the prediction that many IclR-family TFs bind three-box motifs, where one pair of boxes corresponds to the first variant of the motif consensus, and the other pair, to the second variant, suggests that alternative dimerization is possible for IclR-family TFs. This hypothesis requires experimental validation.

We demonstrate that site positioning apparently follows the length of the DNA turn. The majority of site centers are positioned between –20 to –80 nt upstream of the gene start, and in this 60–nt zone the probability of site positioning distinctly oscillates, with the distance between the preferred positions approximately corresponding to one DNA turn. We also have observed that TFs from more than half of the studied orthologous groups bind tandem sites with 18–22 (mainly 19–21) nucleotides between their centers. This distance seems to be optimal for the tetramer binding of the IclR-family TFs, with dimers facing the same side of DNA.

We predict protein–DNA contacts by the analysis of correlations between amino acids of DNA-binding motifs of TFs and nucleotides of their binding sites. The correlation analysis shows that, despite differences in the motif structure, the majority of interacting positions and predicted protein–DNA contacts are similar in both studied groups and conform well to existing experimental data, as well as to previously described general protein–DNA interaction trends.

We have also reconstructed regulons for the IclR-family TFs and analyzed their content, identifying co-occurences between the regulated COGs. IclR-family regulogs vary in size and content, and those COGs that are most frequently present and associated with each other are involved in the metabolism and transport of aromatic compounds and sugars or sugar acids.

## Data Availability Statement

The original contributions presented in the study are included in the article/Supplementary Material, further inquiries can be directed to the corresponding author/s.

## Author Contributions

IS and MG conceived the study, analyzed the results, wrote and revised the manuscript, and read and approved the submitted version. IS designed and performed the comparative analysis and visualized results. Both authors contributed to the article and approved the submitted version.

## Conflict of Interest

The authors declare that the research was conducted in the absence of any commercial or financial relationships that could be construed as a potential conflict of interest.

## Footnotes

1 https://uhasselt.shinyapps.io/shiny-biclust/

2 https://regprecise.lbl.gov/collection_tffam.jsp?tffamily_id=31
