# Supplementary material for: Comparative Analysis of the IclR-Family of Bacterial Transcription Factors and Their DNA-Binding Motifs: Structure, Positioning, Co-Evolution, Regulon Content
Source: Front Microbiol. 2021 Jun 10;12:675815. doi: 10.3389/fmicb.2021.675815 (PMC8222616; doi:10.3389/fmicb.2021.675815)
Supplement: Supplementary file 5 [file Data_Sheet_1.docx]

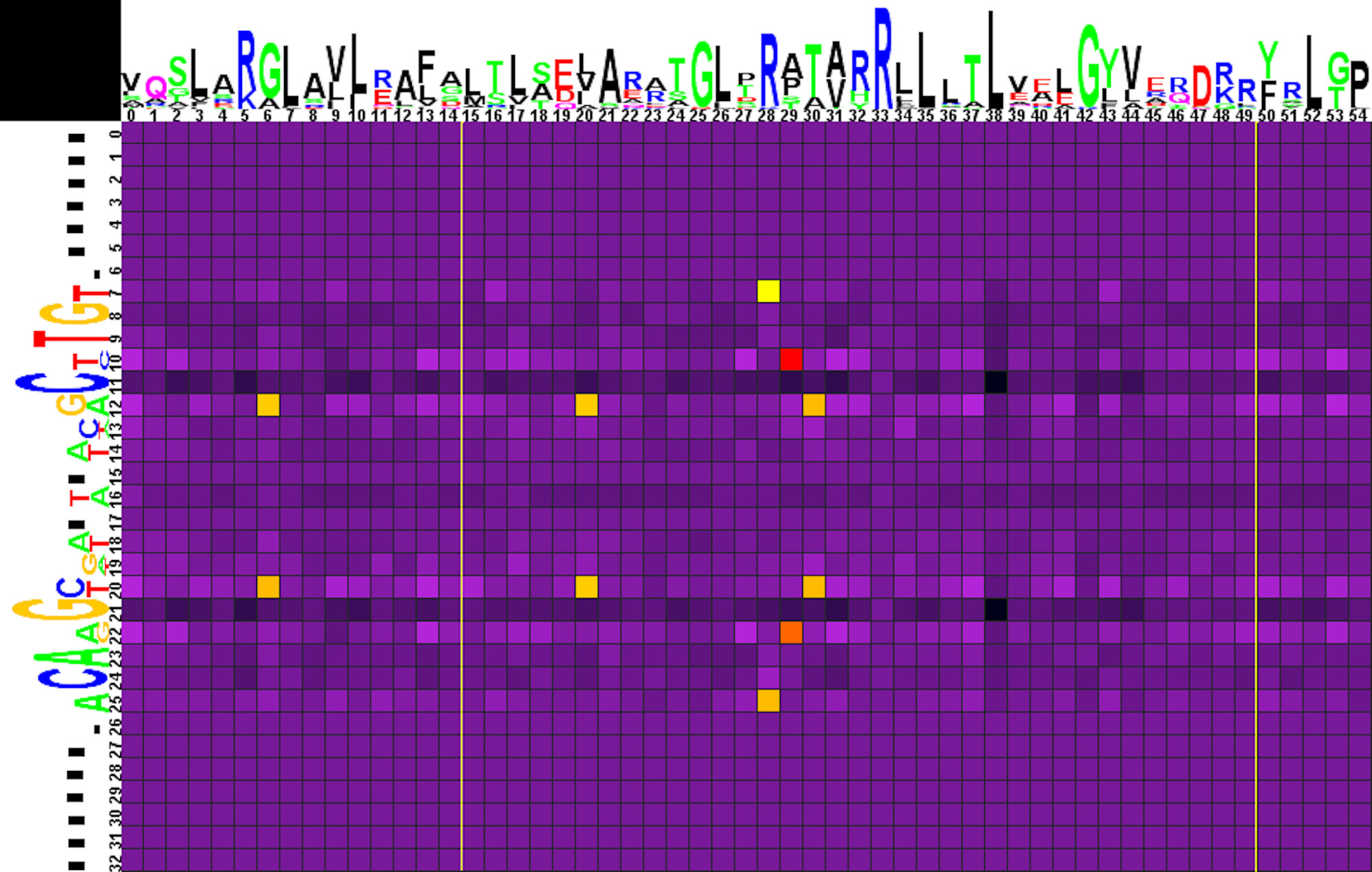


**Figure S1. Heat map of correlations between amino acids and nucleotides for TGT-11-ACA subgroup (group 1) TFs and their binding sites.**

Sequence logos of HTH DNA-binding domains and their binding sites are shown on the top and to the left of the heat map, respectively. The total height of symbols at each position reflects the positional information content, whereas the height of individual symbols is proportional to the positional amino acid or nucleotide frequencies. The correlation scores are color-coded from yellow to red for amino acid-nucleotide pairs with statistical significance of correlation exceeding an automatically defined threshold (with red assigned for the most correlated pair). The violet-black palette is used for other pairs. Yellow lines denote positions where gaps have been removed from the amino acid alignment.


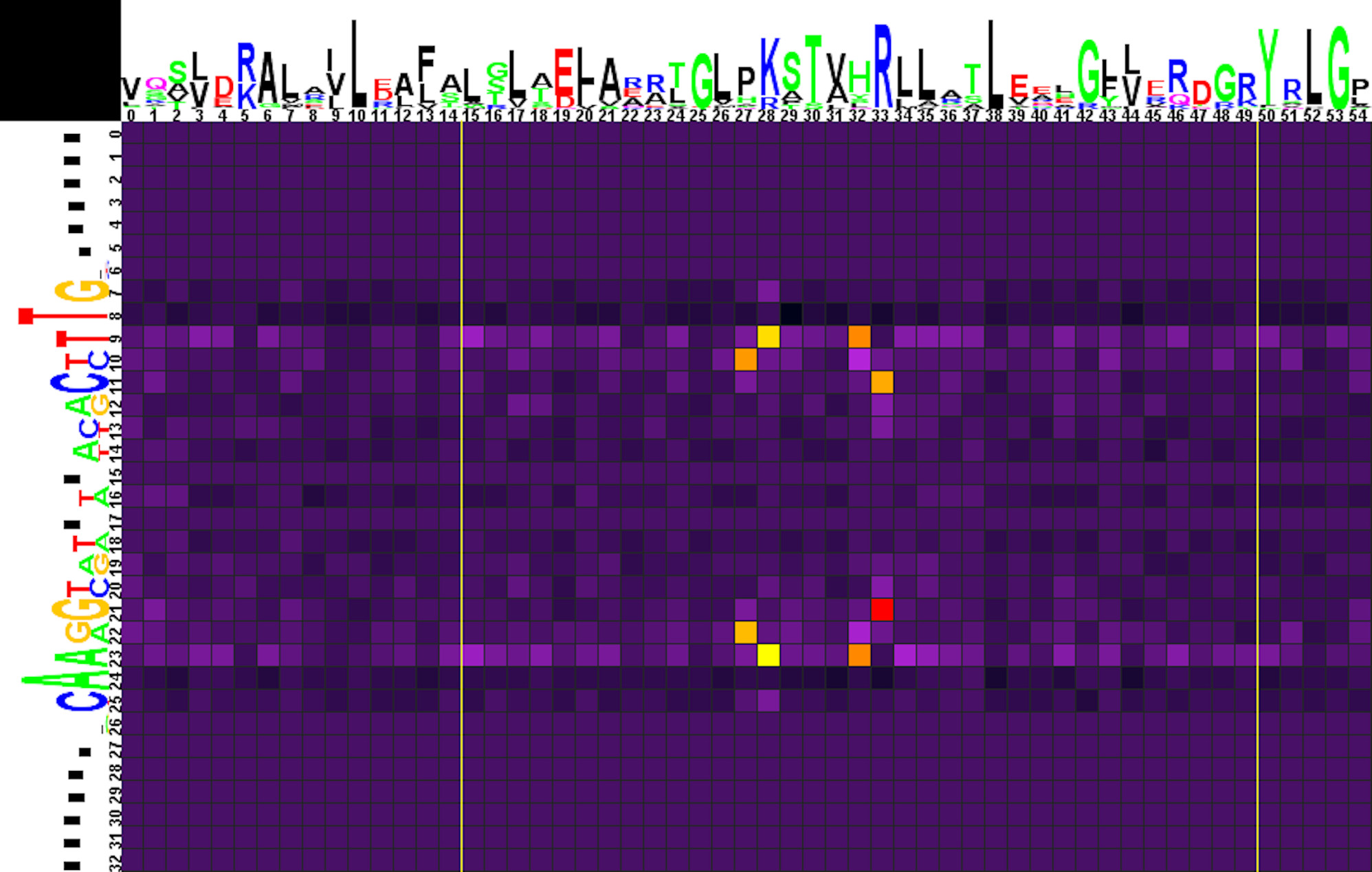


**Figure S2. Heat map of correlations between amino acids and nucleotides for GTT-11-AAC subgroup (group 1) TFs and their binding sites.**

Notation as in Figure S1.


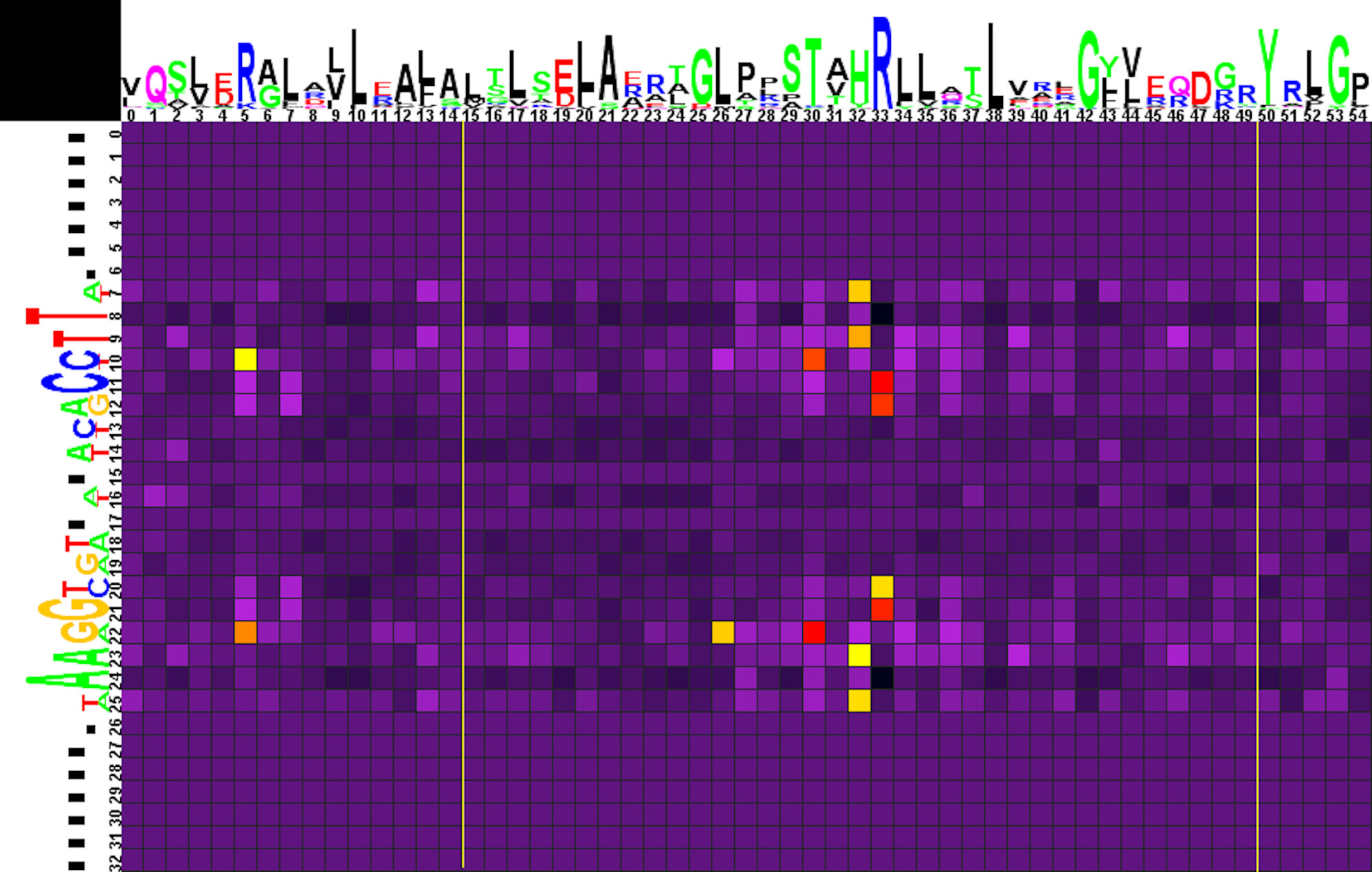


**Figure S3. Heat map of correlations between amino acids and nucleotides for WTT-11-AAW subgroup (group 1) TFs and their binding sites.**

Notation as in Figure S1.


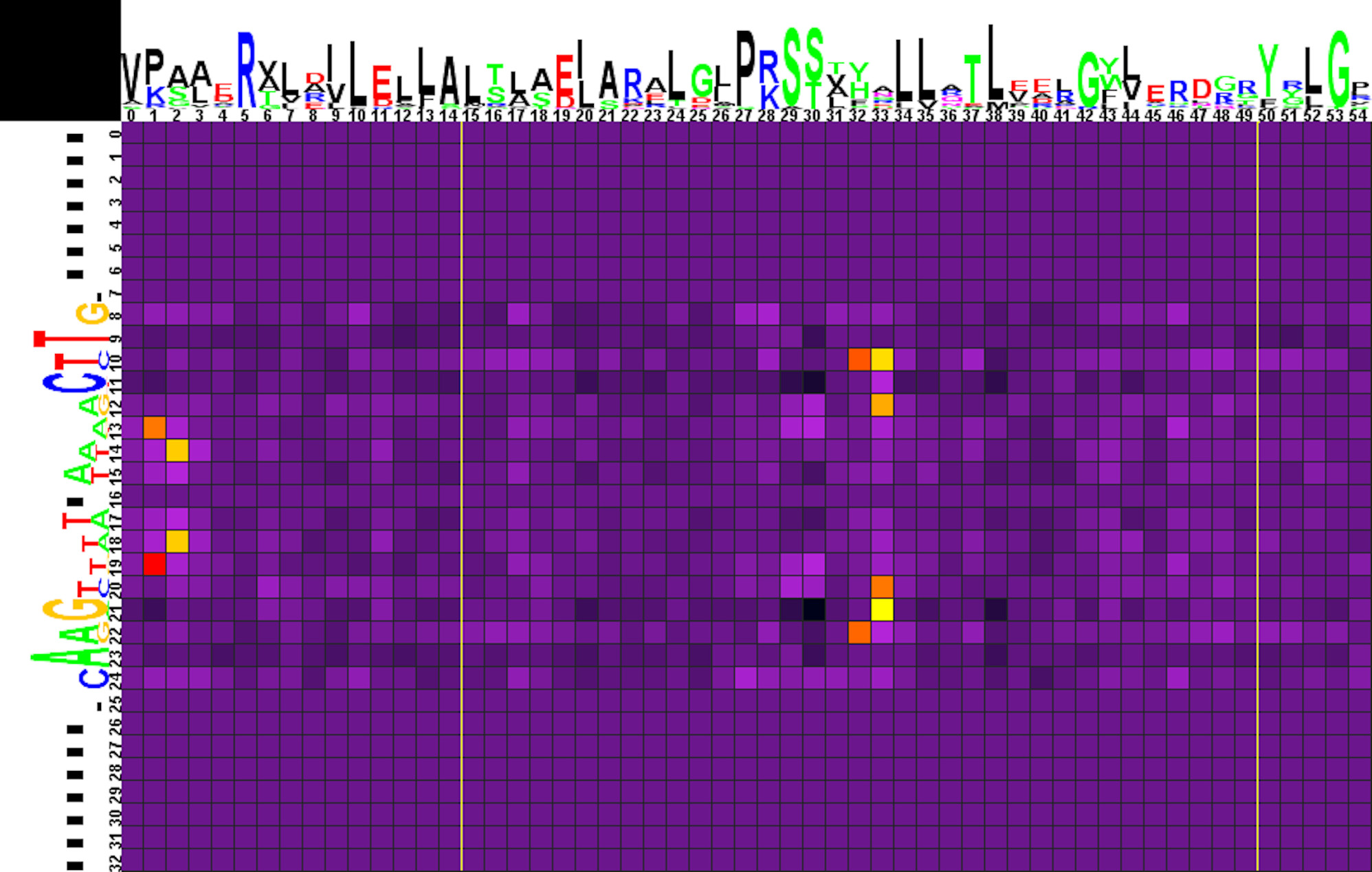


**Figure S4. Heat map of correlations between amino acids and nucleotides for NGT-12-ACN subgroup (group 1) TFs and their binding sites.**

Notation as in Figure S1.


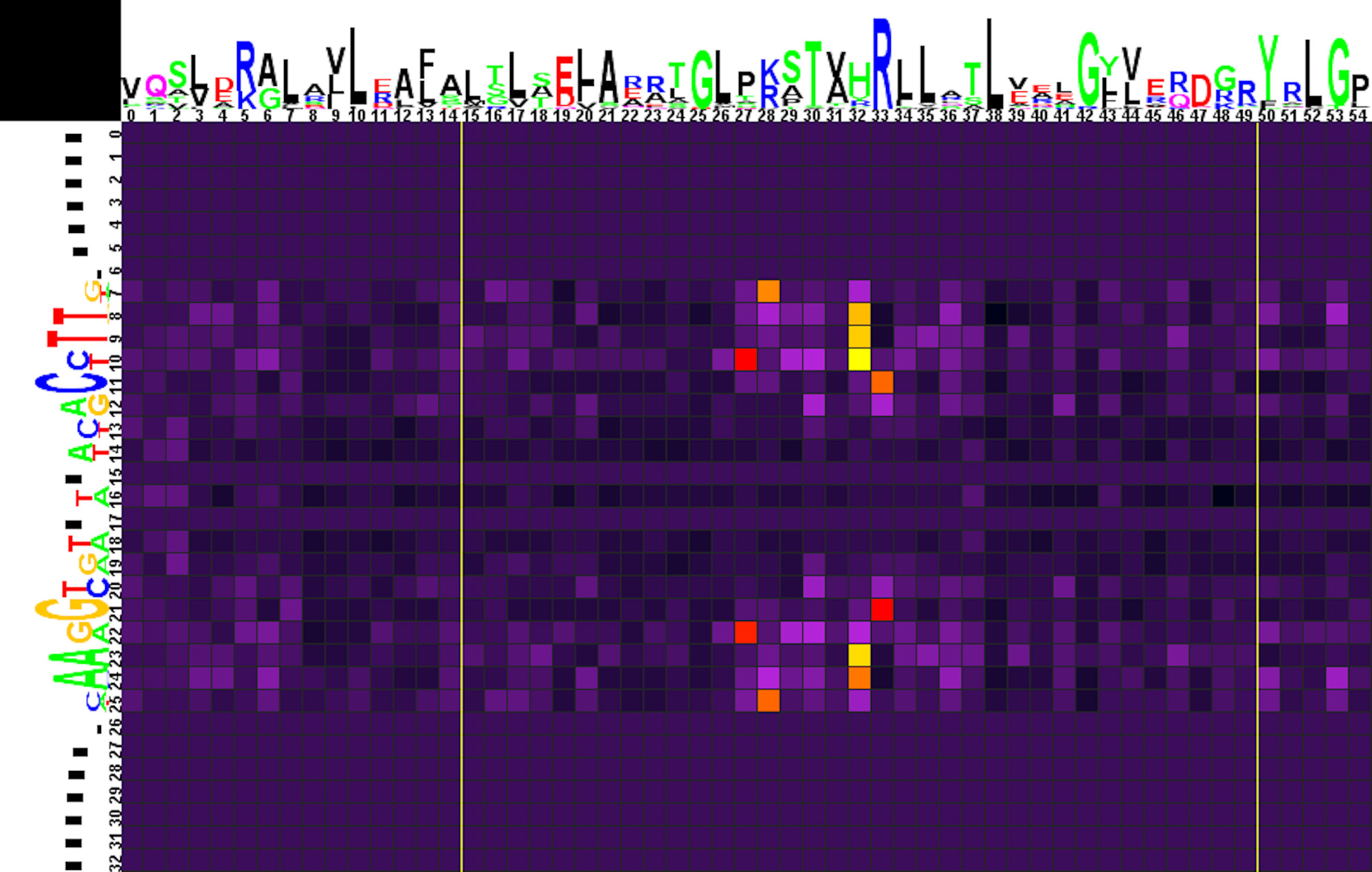


**Figure S5. Heat map of correlations between amino acids and nucleotides for all odd-length (group 1) binding sites and TFs.**

Notation as in Figure S1.
